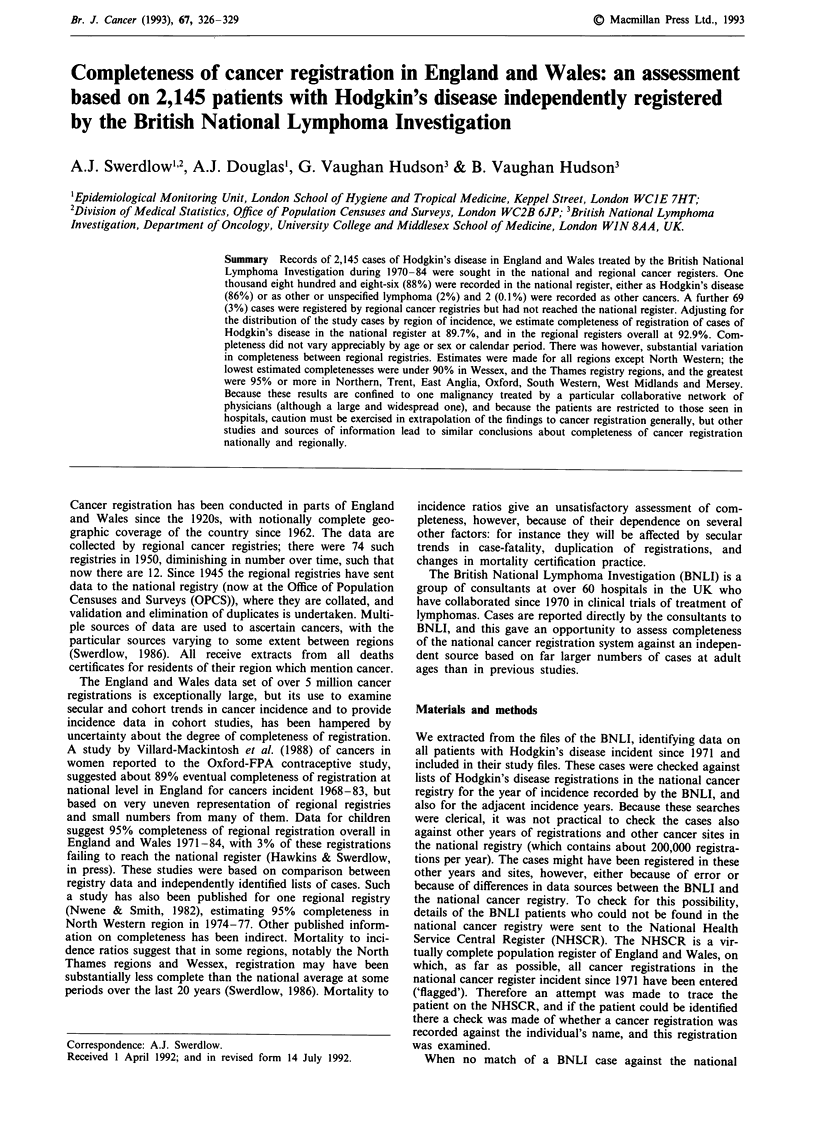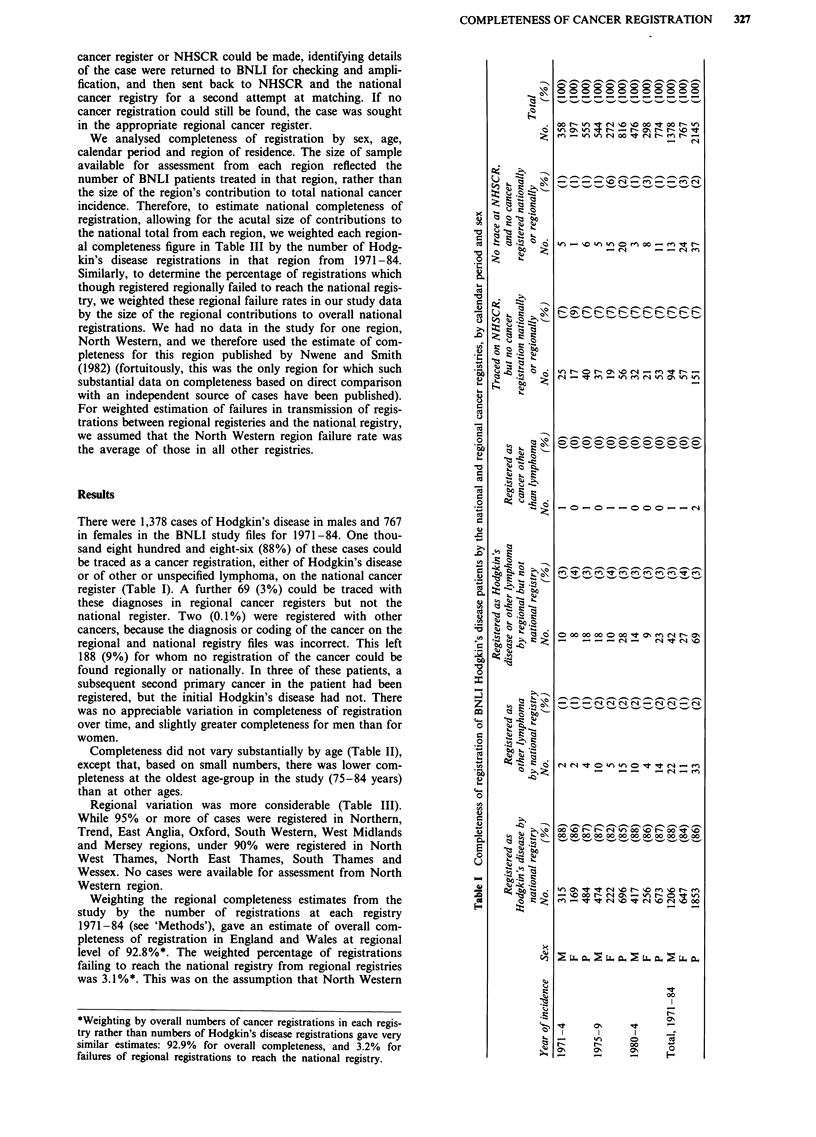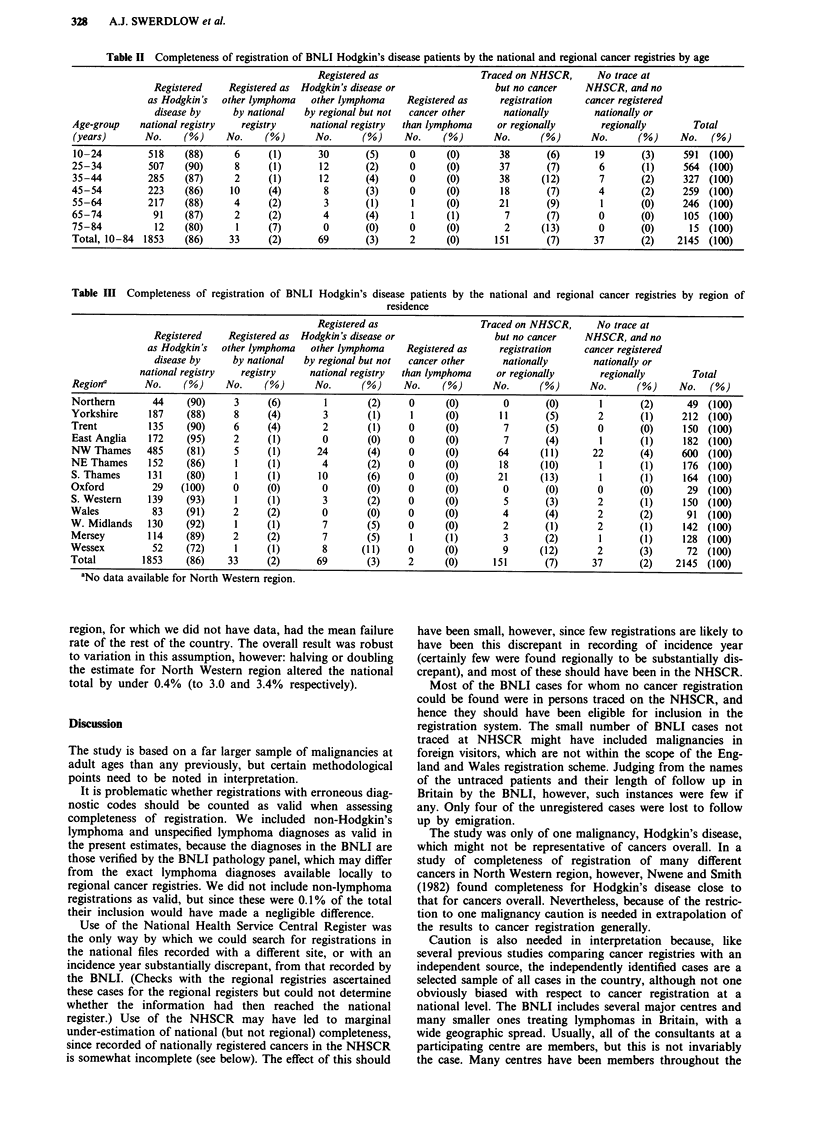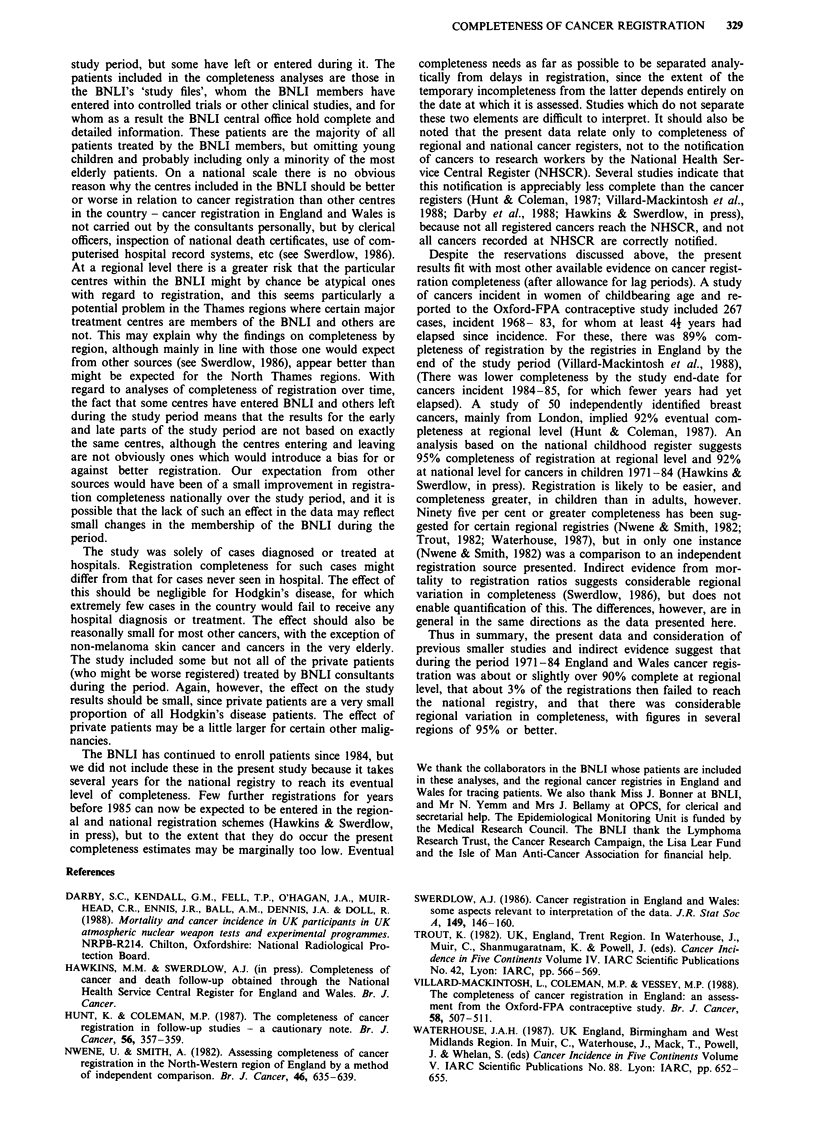# Completeness of cancer registration in England and Wales: an assessment based on 2,145 patients with Hodgkin's disease independently registered by the British National Lymphoma Investigation.

**DOI:** 10.1038/bjc.1993.60

**Published:** 1993-02

**Authors:** A. J. Swerdlow, A. J. Douglas, G. Vaughan Hudson, B. Vaughan Hudson

**Affiliations:** Epidemiological Monitoring Unit, London School of Hygiene and Tropical Medicine, UK.

## Abstract

Records of 2,145 cases of Hodgkin's disease in England and Wales treated by the British National Lymphoma Investigation during 1970-84 were sought in the national and regional cancer registers. One thousand eight hundred and eight-six (88%) were recorded in the national register, either as Hodgkin's disease (86%) or as other or unspecified lymphoma (2%) and 2 (0.1%) were recorded as other cancers. A further 69 (3%) cases were registered by regional cancer registries but had not reached the national register. Adjusting for the distribution of the study cases by region of incidence, we estimate completeness of registration of cases of Hodgkin's disease in the national register at 89.7%, and in the regional registers overall at 92.9%. Completeness did not vary appreciably by age or sex or calendar period. There was however, substantial variation in completeness between regional registries. Estimates were made for all regions except North Western; the lowest estimated completeness were under 90% in Wessex, and the Thames registry regions, and the greatest were 95% or more in Northern, Trent, East Anglia, Oxford, South Western, West Midlands and Mersey. Because these results are confined to one malignancy treated by a particular collaborative network of physicians (although a large and widespread one), and because the patients are restricted to those seen in hospitals, caution must be exercised in extrapolation of the findings to cancer registration generally, but other studies and sources of information lead to similar conclusions about completeness of cancer registration nationally and regionally.


Br. J. Cancer (1993), 67, 326-329 © Macmillan Press Ltd., 1993

### Completeness of cancer registration in England and Wales: an assessment based on 2,145 patients with Hodgkin's disease independently registered by the British National Lymphoma Investigation

A.J. Swerdlow[1,2], A.J. Douglas[1], G. Vaughan Hudson[3] & B. Vaughan Hudson[3]

[1]*Epidemiological Monitoring Unit, London School of Hygiene and Tropical Medicine, Keppel Street, London WC1E 7HT;* [2]*Division of Medical Statistics, Office of Population Censuses and Surveys, London WC2B 6JP;* [3]*British National Lymphoma Investigation, Department of Oncology, University College and Middlesex School of Medicine, London W1N 8AA, UK.*

**Summary** Records of 2,145 cases of Hodgkin's disease in England and Wales treated by the British National Lymphoma Investigation during 1970-84 were sought in the national and regional cancer registers. One thousand eight hundred and eight-six (88%) were recorded in the national register, either as Hodgkin's disease (86%) or as other or unspecified lymphoma (2%) and 2 (0.1%) were recorded as other cancers. A further 69 (3%) cases were registered by regional cancer registries but had not reached the national register. Adjusting for the distribution of the study cases by region of incidence, we estimate completeness of registration of cases of Hodgkin's disease in the national register at 89.7%, and in the regional registers overall at 92.9%. Completeness did not vary appreciably by age or sex or calendar period. There was however, substantial variation in completeness between regional registries. Estimates were made for all regions except North Western; the lowest estimated completenesses were under 90% in Wessex, and the Thames registry regions, and the greatest were 95% or more in Northern, Trent, East Anglia, Oxford, South Western, West Midlands and Mersey. Because these results are confined to one malignancy treated by a particular collaborative network of physicians (although a large and widespread one), and because the patients are restricted to those seen in hospitals, caution must be exercised in extrapolation of the findings to cancer registration generally, but other studies and sources of information lead to similar conclusions about completeness of cancer registration nationally and regionally.

Cancer registration has been conducted in parts of England and Wales since the 1920s, with notionally complete geographic coverage of the country since 1962. The data are collected by regional cancer registries; there were 74 such registries in 1950, diminishing in number over time, such that now there are 12. Since 1945 the regional registries have sent data to the national registry (now at the Office of Population Censuses and Surveys (OPCS)), where they are collated, and validation and elimination of duplicates is undertaken. Multiple sources of data are used to ascertain cancers, with the particular sources varying to some extent between regions (Swerdlow, 1986). All receive extracts from all deaths certificates for residents of their region which mention cancer.

The England and Wales data set of over 5 million cancer registrations is exceptionally large, but its use to examine secular and cohort trends in cancer incidence and to provide incidence data in cohort studies, has been hampered by uncertainty about the degree of completeness of registration. A study by Villard-Mackintosh *et al.* (1988) of cancers in women reported to the Oxford-FPA contraceptive study, suggested about 89% eventual completeness of registration at national level in England for cancers incident 1968-83, but based on very uneven representation of regional registries and small numbers from many of them. Data for children suggest 95% completeness of regional registration overall in England and Wales 1971-84, with 3% of these registrations failing to reach the national register (Hawkins & Swerdlow, in press). These studies were based on comparison between registry data and independently identified lists of cases. Such a study has also been published for one regional registry (Nwene & Smith, 1982), estimating 95% completeness in North Western region in 1974-77. Other published information on completeness has been indirect. Mortality to incidence ratios suggest that in some regions, notably the North Thames regions and Wessex, registration may have been substantially less complete than the national average at some periods over the last 20 years (Swerdlow, 1986). Mortality to incidence ratios give an unsatisfactory assessment of completeness, however, because of their dependence on several other factors: for instance they will be affected by secular trends in case-fatality, duplication of registrations, and changes in mortality certification practice.

The British National Lymphoma Investigation (BNLI) is a group of consultants at over 60 hospitals in the UK who have collaborated since 1970 in clinical trials of treatment of lymphomas. Cases are reported directly by the consultants to BNLI, and this gave an opportunity to assess completeness of the national cancer registration system against an independent source based on far larger numbers of cases at adult ages than in previous studies.

### Materials and methods

We extracted from the files of the BNLI, identifying data on all patients with Hodgkin's disease incident since 1971 and included in their study files. These cases were checked against lists of Hodgkin's disease registrations in the national cancer registry for the year of incidence recorded by the BNLI, and also for the adjacent incidence years. Because these searches were clerical, it was not practical to check the cases also against other years of registrations and other cancer sites in the national registry (which contains about 200,000 registrations per year). The cases might have been registered in these other years and sites, however, either because of error or because of differences in data sources between the BNLI and the national cancer registry. To check for this possibility, details of the BNLI patients who could not be found in the national cancer registry were sent to the National Health Service Central Register (NHSCR). The NHSCR is a virtually complete population register of England and Wales, on which, as far as possible, all cancer registrations in the national cancer register incident since 1971 have been entered ('flagged'). Therefore an attempt was made to trace the patient on the NHSCR, and if the patient could be identified there a check was made of whether a cancer registration was recorded against the individual's name, and this registration was examined.

When no match of a BNLI case against the national

Correspondence: A.J. Swerdlow.
Received 1 April 1992; and in revised form 14 July 1992.

cancer register or NHSCR could be made, identifying details of the case were returned to BNLI for checking and amplification, and then sent back to NHSCR and the national cancer registry for a second attempt at matching. If no cancer registration could still be found, the case was sought in the appropriate regional cancer register.

We analysed completeness of registration by sex, age, calendar period and region of residence. The size of sample available for assessment from each region reflected the number of BNLI patients treated in that region, rather than the size of the region's contribution to total national cancer incidence. Therefore, to estimate national completeness of registration, allowing for the acutal size of contributions to the national total from each region, we weighted each regional completeness figure in Table III by the number of Hodgkin's disease registrations in that region from 1971–84. Similarly, to determine the percentage of registrations which though registered regionally failed to reach the national registry, we weighted these regional failure rates in our study data by the size of the regional contributions to overall national registrations. We had no data in the study for one region, North Western, and we therefore used the estimate of completeness for this region published by Nwene and Smith (1982) (fortuitously, this was the only region for which such substantial data on completeness based on direct comparison with an independent source of cases have been published). For weighted estimation of failures in transmission of registrations between regional registeries and the national registry, we assumed that the North Western region failure rate was the average of those in all other registries.

### Results

There were 1,378 cases of Hodgkin's disease in males and 767 in females in the BNLI study files for 1971–84. One thousand eight hundred and eight-six (88%) of these cases could be traced as a cancer registration, either of Hodgkin's disease or of other or unspecified lymphoma, on the national cancer register (Table I). A further 69 (3%) could be traced with these diagnoses in regional cancer registers but not the national register. Two (0.1%) were registered with other cancers, because the diagnosis or coding of the cancer on the regional and national registry files was incorrect. This left 188 (9%) for whom no registration of the cancer could be found regionally or nationally. In three of these patients, a subsequent second primary cancer in the patient had been registered, but the initial Hodgkin's disease had not. There was no appreciable variation in completeness of registration over time, and slightly greater completeness for men than for women.

Completeness did not vary substantially by age (Table II), except that, based on small numbers, there was lower completeness at the oldest age-group in the study (75–84 years) than at other ages.

Regional variation was more considerable (Table III). While 95% or more of cases were registered in Northern, Trend, East Anglia, Oxford, South Western, West Midlands and Mersey regions, under 90% were registered in North West Thames, North East Thames, South Thames and Wessex. No cases were available for assessment from North Western region.

Weighting the regional completeness estimates from the study by the number of registrations at each registry 1971–84 (see 'Methods'), gave an estimate of overall completeness of registration in England and Wales at regional level of 92.8%*. The weighted percentage of registrations failing to reach the national registry from regional registries was 3.1%*. This was on the assumption that North Western

*Weighting by overall numbers of cancer registrations in each registry rather than numbers of Hodgkin's disease registrations gave very similar estimates: 92.9% for overall completeness, and 3.2% for failures of regional registrations to reach the national registry.

**Table I** Completeness of registration of BNLI Hodgkin's disease patients by the national and regional cancer registries, by calendar period and sex

| Year of incidence | Sex | Registered as Hodgkin's disease by national registry No. | (%) | Registered as other lymphoma by national registry No. | (%) | Registered as Hodgkin's disease or other lymphoma by regional but not national registry No. | (%) | Registered as cancer other than lymphoma No. | (%) | Traced on NHSCR, but no cancer registration nationally or regionally No. | (%) | No trace at NHSCR, and no cancer registered nationally or regionally No. | (%) | Total No. | (%) |
|---|---|---|---|---|---|---|---|---|---|---|---|---|---|---|---|
| 1971–4 | M | 315 | (88) | 2 | (1) | 10 | (3) | 1 | (0) | 25 | (7) | 5 | (1) | 358 | (100) |
| | F | 169 | (86) | 2 | (1) | 8 | (4) | 0 | (0) | 17 | (9) | 1 | (1) | 197 | (100) |
| | P | 484 | (87) | 4 | (1) | 18 | (3) | 1 | (0) | 40 | (7) | 6 | (1) | 555 | (100) |
| 1975–9 | M | 474 | (87) | 10 | (2) | 18 | (3) | 0 | (0) | 37 | (7) | 5 | (1) | 544 | (100) |
| | F | 222 | (82) | 5 | (2) | 10 | (4) | 1 | (0) | 19 | (7) | 15 | (6) | 272 | (100) |
| | P | 696 | (85) | 15 | (2) | 28 | (3) | 1 | (0) | 56 | (7) | 20 | (2) | 816 | (100) |
| 1980–4 | M | 417 | (88) | 10 | (2) | 14 | (3) | 0 | (0) | 32 | (7) | 3 | (1) | 476 | (100) |
| | F | 256 | (86) | 4 | (1) | 9 | (3) | 0 | (0) | 21 | (7) | 8 | (3) | 298 | (100) |
| | P | 673 | (87) | 14 | (2) | 23 | (3) | 0 | (0) | 53 | (7) | 11 | (1) | 774 | (100) |
| Total, 1971–84 | M | 1206 | (88) | 22 | (2) | 42 | (3) | 1 | (0) | 94 | (7) | 13 | (1) | 1378 | (100) |
| | F | 647 | (84) | 11 | (1) | 27 | (4) | 1 | (0) | 57 | (7) | 24 | (3) | 767 | (100) |
| | P | 1853 | (86) | 33 | (2) | 69 | (3) | 2 | (0) | 151 | (7) | 37 | (2) | 2145 | (100) |

**Table II** Completeness of registration of BNLI Hodgkin's disease patients by the national and regional cancer registries by age

| *Age-group (years)* | *Registered as Hodgkin's disease by national registry* | | *Registered as other lymphoma by national registry* | | *Registered as Hodgkin's disease or other lymphoma by regional but not national registry* | | *Registered as cancer other than lymphoma* | | *Traced on NHSCR, but no cancer registration nationally or regionally* | | *No trace at NHSCR, and no cancer registered nationally or regionally* | | *Total* | |
|---|---|---|---|---|---|---|---|---|---|---|---|---|---|---|
| | *No.* | *(%)* | *No.* | *(%)* | *No.* | *(%)* | *No.* | *(%)* | *No.* | *(%)* | *No.* | *(%)* | *No.* | *(%)* |
| 10–24 | 518 | (88) | 6 | (1) | 30 | (5) | 0 | (0) | 38 | (6) | 19 | (3) | 591 | (100) |
| 25–34 | 507 | (90) | 8 | (1) | 12 | (2) | 0 | (0) | 37 | (7) | 6 | (1) | 564 | (100) |
| 35–44 | 285 | (87) | 2 | (1) | 12 | (4) | 0 | (0) | 38 | (12) | 7 | (2) | 327 | (100) |
| 45–54 | 223 | (86) | 10 | (4) | 8 | (3) | 0 | (0) | 18 | (7) | 4 | (2) | 259 | (100) |
| 55–64 | 217 | (88) | 4 | (2) | 3 | (1) | 1 | (0) | 21 | (9) | 1 | (0) | 246 | (100) |
| 65–74 | 91 | (87) | 2 | (2) | 4 | (4) | 1 | (1) | 7 | (7) | 0 | (0) | 105 | (100) |
| 75–84 | 12 | (80) | 1 | (7) | 0 | (0) | 0 | (0) | 2 | (13) | 0 | (0) | 15 | (100) |
| Total, 10–84 | 1853 | (86) | 33 | (2) | 69 | (3) | 2 | (0) | 151 | (7) | 37 | (2) | 2145 | (100) |

**Table III** Completeness of registration of BNLI Hodgkin's disease patients by the national and regional cancer registries by region of residence

| *Region*[a] | *Registered as Hodgkin's disease by national registry* | | *Registered as other lymphoma by national registry* | | *Registered as Hodgkin's disease or other lymphoma by regional but not national registry* | | *Registered as cancer other than lymphoma* | | *Traced on NHSCR, but no cancer registration nationally or regionally* | | *No trace at NHSCR, and no cancer registered nationally or regionally* | | *Total* | |
|---|---|---|---|---|---|---|---|---|---|---|---|---|---|---|
| | *No.* | *(%)* | *No.* | *(%)* | *No.* | *(%)* | *No.* | *(%)* | *No.* | *(%)* | *No.* | *(%)* | *No.* | *(%)* |
| Northern | 44 | (90) | 3 | (6) | 1 | (2) | 0 | (0) | 0 | (0) | 1 | (2) | 49 | (100) |
| Yorkshire | 187 | (88) | 8 | (4) | 3 | (1) | 1 | (0) | 11 | (5) | 2 | (1) | 212 | (100) |
| Trent | 135 | (90) | 6 | (4) | 2 | (1) | 0 | (0) | 7 | (5) | 0 | (0) | 150 | (100) |
| East Anglia | 172 | (95) | 2 | (1) | 0 | (0) | 0 | (0) | 7 | (4) | 1 | (1) | 182 | (100) |
| NW Thames | 485 | (81) | 5 | (1) | 24 | (4) | 0 | (0) | 64 | (11) | 22 | (4) | 600 | (100) |
| NE Thames | 152 | (86) | 1 | (1) | 4 | (2) | 0 | (0) | 18 | (10) | 1 | (1) | 176 | (100) |
| S. Thames | 131 | (80) | 1 | (1) | 10 | (6) | 0 | (0) | 21 | (13) | 1 | (1) | 164 | (100) |
| Oxford | 29 | (100) | 0 | (0) | 0 | (0) | 0 | (0) | 0 | (0) | 0 | (0) | 29 | (100) |
| S. Western | 139 | (93) | 1 | (1) | 3 | (2) | 0 | (0) | 5 | (3) | 2 | (1) | 150 | (100) |
| Wales | 83 | (91) | 2 | (2) | 0 | (0) | 0 | (0) | 4 | (4) | 2 | (2) | 91 | (100) |
| W. Midlands | 130 | (92) | 1 | (1) | 7 | (5) | 0 | (0) | 2 | (1) | 2 | (1) | 142 | (100) |
| Mersey | 114 | (89) | 2 | (2) | 7 | (5) | 1 | (1) | 3 | (2) | 1 | (1) | 128 | (100) |
| Wessex | 52 | (72) | 1 | (1) | 8 | (11) | 0 | (0) | 9 | (12) | 2 | (3) | 72 | (100) |
| Total | 1853 | (86) | 33 | (2) | 69 | (3) | 2 | (0) | 151 | (7) | 37 | (2) | 2145 | (100) |

[a]No data available for North Western region.

region, for which we did not have data, had the mean failure rate of the rest of the country. The overall result was robust to variation in this assumption, however: halving or doubling the estimate for North Western region altered the national total by under 0.4% (to 3.0 and 3.4% respectively).

### Discussion

The study is based on a far larger sample of malignancies at adult ages than any previously, but certain methodological points need to be noted in interpretation.

It is problematic whether registrations with erroneous diagnostic codes should be counted as valid when assessing completeness of registration. We included non-Hodgkin's lymphoma and unspecified lymphoma diagnoses as valid in the present estimates, because the diagnoses in the BNLI are those verified by the BNLI pathology panel, which may differ from the exact lymphoma diagnoses available locally to regional cancer registries. We did not include non-lymphoma registrations as valid, but since these were 0.1% of the total their inclusion would have made a negligible difference.

Use of the National Health Service Central Register was the only way by which we could search for registrations in the national files recorded with a different site, or with an incidence year substantially discrepant, from that recorded by the BNLI. (Checks with the regional registries ascertained these cases for the regional registers but could not determine whether the information had then reached the national register.) Use of the NHSCR may have led to marginal under-estimation of national (but not regional) completeness, since recorded of nationally registered cancers in the NHSCR is somewhat incomplete (see below). The effect of this should have been small, however, since few registrations are likely to have been this discrepant in recording of incidence year (certainly few were found regionally to be substantially discrepant), and most of these should have been in the NHSCR.

Most of the BNLI cases for whom no cancer registration could be found were in persons traced on the NHSCR, and hence they should have been eligible for inclusion in the registration system. The small number of BNLI cases not traced at NHSCR might have included malignancies in foreign visitors, which are not within the scope of the England and Wales registration scheme. Judging from the names of the untraced patients and their length of follow up in Britain by the BNLI, however, such instances were few if any. Only four of the unregistered cases were lost to follow up by emigration.

The study was only of one malignancy, Hodgkin's disease, which might not be representative of cancers overall. In a study of completeness of registration of many different cancers in North Western region, however, Nwene and Smith (1982) found completeness for Hodgkin's disease close to that for cancers overall. Nevertheless, because of the restriction to one malignancy caution is needed in extrapolation of the results to cancer registration generally.

Caution is also needed in interpretation because, like several previous studies comparing cancer registries with an independent source, the independently identified cases are a selected sample of all cases in the country, although not one obviously biased with respect to cancer registration at a national level. The BNLI includes several major centres and many smaller ones treating lymphomas in Britain, with a wide geographic spread. Usually, all of the consultants at a participating centre are members, but this is not invariably the case. Many centres have been members throughout the study period, but some have left or entered during it. The patients included in the completeness analyses are those in the BNLI's 'study files', whom the BNLI members have entered into controlled trials or other clinical studies, and for whom as a result the BNLI central office hold complete and detailed information. These patients are the majority of all patients treated by the BNLI members, but omitting young children and probably including only a minority of the most elderly patients. On a national scale there is no obvious reason why the centres included in the BNLI should be better or worse in relation to cancer registration than other centres in the country – cancer registration in England and Wales is not carried out by the consultants personally, but by clerical officers, inspection of national death certificates, use of computerised hospital record systems, etc (see Swerdlow, 1986). At a regional level there is a greater risk that the particular centres within the BNLI might by chance be atypical ones with regard to registration, and this seems particularly a potential problem in the Thames regions where certain major treatment centres are members of the BNLI and others are not. This may explain why the findings on completeness by region, although mainly in line with those one would expect from other sources (see Swerdlow, 1986), appear better than might be expected for the North Thames regions. With regard to analyses of completeness of registration over time, the fact that some centres have entered BNLI and others left during the study period means that the results for the early and late parts of the study period are not based on exactly the same centres, although the centres entering and leaving are not obviously ones which would introduce a bias for or against better registration. Our expectation from other sources would have been of a small improvement in registration completeness nationally over the study period, and it is possible that the lack of such an effect in the data may reflect small changes in the membership of the BNLI during the period.

The study was solely of cases diagnosed or treated at hospitals. Registration completeness for such cases might differ from that for cases never seen in hospital. The effect of this should be negligible for Hodgkin's disease, for which extremely few cases in the country would fail to receive any hospital diagnosis or treatment. The effect should also be reasonally small for most other cancers, with the exception of non-melanoma skin cancer and cancers in the very elderly. The study included some but not all of the private patients (who might be worse registered) treated by BNLI consultants during the period. Again, however, the effect on the study results should be small, since private patients are a very small proportion of all Hodgkin's disease patients. The effect of private patients may be a little larger for certain other malignancies.

The BNLI has continued to enroll patients since 1984, but we did not include these in the present study because it takes several years for the national registry to reach its eventual level of completeness. Few further registrations for years before 1985 can now be expected to be entered in the regional and national registration schemes (Hawkins & Swerdlow, in press), but to the extent that they do occur the present completeness estimates may be marginally too low. Eventual completeness needs as far as possible to be separated analytically from delays in registration, since the extent of the temporary incompleteness from the latter depends entirely on the date at which it is assessed. Studies which do not separate these two elements are difficult to interpret. It should also be noted that the present data relate only to completeness of regional and national cancer registers, not to the notification of cancers to research workers by the National Health Service Central Register (NHSCR). Several studies indicate that this notification is appreciably less complete than the cancer registers (Hunt & Coleman, 1987; Villard-Mackintosh *et al.*, 1988; Darby *et al.*, 1988; Hawkins & Swerdlow, in press), because not all registered cancers reach the NHSCR, and not all cancers recorded at NHSCR are correctly notified.

Despite the reservations discussed above, the present results fit with most other available evidence on cancer registration completeness (after allowance for lag periods). A study of cancers incident in women of childbearing age and reported to the Oxford-FPA contraceptive study included 267 cases, incident 1968– 83, for whom at least 4½ years had elapsed since incidence. For these, there was 89% completeness of registration by the registries in England by the end of the study period (Villard-Mackintosh *et al.*, 1988), (There was lower completeness by the study end-date for cancers incident 1984–85, for which fewer years had yet elapsed). A study of 50 independently identified breast cancers, mainly from London, implied 92% eventual completeness at regional level (Hunt & Coleman, 1987). An analysis based on the national childhood register suggests 95% completeness of registration at regional level and 92% at national level for cancers in children 1971–84 (Hawkins & Swerdlow, in press). Registration is likely to be easier, and completeness greater, in children than in adults, however. Ninety five per cent or greater completeness has been suggested for certain regional registries (Nwene & Smith, 1982; Trout, 1982; Waterhouse, 1987), but in only one instance (Nwene & Smith, 1982) was a comparison to an independent registration source presented. Indirect evidence from mortality to registration ratios suggests considerable regional variation in completeness (Swerdlow, 1986), but does not enable quantification of this. The differences, however, are in general in the same directions as the data presented here.

Thus in summary, the present data and consideration of previous smaller studies and indirect evidence suggest that during the period 1971–84 England and Wales cancer registration was about or slightly over 90% complete at regional level, that about 3% of the registrations then failed to reach the national registry, and that there was considerable regional variation in completeness, with figures in several regions of 95% or better.

We thank the collaborators in the BNLI whose patients are included in these analyses, and the regional cancer registries in England and Wales for tracing patients. We also thank Miss J. Bonner at BNLI, and Mr N. Yemm and Mrs J. Bellamy at OPCS, for clerical and secretarial help. The Epidemiological Monitoring Unit is funded by the Medical Research Council. The BNLI thank the Lymphoma Research Trust, the Cancer Research Campaign, the Lisa Lear Fund and the Isle of Man Anti-Cancer Association for financial help.

### References

DARBY, S.C., KENDALL, G.M., FELL, T.P., O'HAGAN, J.A., MUIRHEAD, C.R., ENNIS, J.R., BALL, A.M., DENNIS, J.A. & DOLL, R. (1988). *Mortality and cancer incidence in UK participants in UK atmospheric nuclear weapon tests and experimental programmes.* NRPB-R214. Chilton, Oxfordshire: National Radiological Protection Board.

HAWKINS, M.M. & SWERDLOW, A.J. (in press). Completeness of cancer and death follow-up obtained through the National Health Service Central Register for England and Wales. *Br. J. Cancer.*

HUNT, K. & COLEMAN, M.P. (1987). The completeness of cancer registration in follow-up studies – a cautionary note. *Br. J. Cancer*, **56**, 357–359.

NWENE, U. & SMITH, A. (1982). Assessing completeness of cancer registration in the North-Western region of England by a method of independent comparison. *Br. J. Cancer*, **46**, 635–639.

SWERDLOW, A.J. (1986). Cancer registration in England and Wales: some aspects relevant to interpretation of the data. *J.R. Stat Soc A*, **149**, 146–160.

TROUT, K. (1982). UK, England, Trent Region. In Waterhouse, J., Muir, C., Shanmugaratnam, K. & Powell, J. (eds). *Cancer Incidence in Five Continents* Volume IV. IARC Scientific Publications No. 42, Lyon: IARC, pp. 566–569.

VILLARD-MACKINTOSH, L., COLEMAN, M.P. & VESSEY, M.P. (1988). The completeness of cancer registration in England: an assessment from the Oxford-FPA contraceptive study. *Br. J. Cancer*, **58**, 507–511.

WATERHOUSE, J.A.H. (1987). UK England, Birmingham and West Midlands Region. In Muir, C., Waterhouse, J., Mack, T., Powell, J. & Whelan, S. (eds) *Cancer Incidence in Five Continents* Volume V. IARC Scientific Publications No. 88. Lyon: IARC, pp. 652–655.